# Effect of Integrated Cognitive Therapy on Hippocampal Functional Connectivity Patterns in Stroke Patients with Cognitive Dysfunction: A Resting-State fMRI Study

**DOI:** 10.1155/2014/962304

**Published:** 2014-12-08

**Authors:** Shanli Yang, Cai Jiang, Haicheng Ye, Jing Tao, Jia Huang, Yanling Gao, Zhicheng Lin, Lidian Chen

**Affiliations:** ^1^Department of Rehabilitation Medicine, Rehabilitation Hospital, Fujian University of Traditional Chinese Medicine, Fujian 350003, China; ^2^Fujian University of Traditional Chinese Medicine, Fujian 350108, China; ^3^Fujian University of Traditional Chinese Medicine, 1 Huatuo Road, Minhou Shangjie, Fuzhou, Fujian 350108, China

## Abstract

*Objective*. This study aimed to identify abnormal hippocampal functional connectivity (FC) following ischemic stroke using resting-state fMRI. We also explored whether abnormal hippocampal FC could be modulated by integrated cognitive therapy and tested whether these alterations were associated with cognitive performance. *Methods*. 18 right-handed cognitively impaired ischemic stroke patients and 18 healty control (HC) subjects were included in this study. Stroke subjects were scanned at baseline and after integrated cognitive therapy, while HCs were only scanned at baseline, to identify regions that show significant correlations with the seed region. Behavioral and cognitive assessments were obtained before each scan. *Results*. During the resting state, we found abnormal hippocampal FC associated with temporal regions, insular cortex, cerebellum, and prefrontal cortex in stroke patients compared to HCs. After integrated cognitive therapy, however, the stroke group showed increased hippocampal FC mainly located in the prefrontal gyrus and the default mode network (DMN). Altered hippocampal FC was associated with cognitive improvement. *Conclusion*. Resting-state fMRI may provide novel insight into the study of functional networks in the brain after stroke. Furthermore, altered hippocampal FC may be a compensatory mechanism for cognitive recovery after ischemic stroke.

## 1. Introduction

Stroke, particularly ischemic stroke, is a leading cause of death and disability in the elderly worldwide [1]. Cognitive dysfunction is considered one of the most common consequences of stroke, often leading to a reduced quality of life, as well as being an economic burden to the patient and community due to increasing medical expenses [2]. Because low frequency fluctuations in resting brain are considered a manifestation of its functional connectivity (FC) [3], probing brain FC is an effective method for detecting disorders associated with dysfunction of its widely distributed neural networks [4]. For example, resting-state functional magnetic resonance imaging (fMRI) studies have used FC to investigate the organization of and changes in functional brain networks, such as those resulting from disease [5].

Considering that the hippocampus is a major brain region associated with learning and memory; changes in hippocampal FC with cortical and subcortical regions have been found in cognitive disorders [6, 7]. Few studies on resting-state FC of stroke patients have been published to date, and research regarding the influence of cognitive training on brain function in cognitively impaired stroke patients using resting-state fMRI is lacking, as most previous studies have concentrated on recovery of motor function [8–10].

Cognitive dysfunction following a stroke can be so complex that one single treatment may not effectively resolve symptoms. Moreover, there are still no US Food and Drug Administration-approved treatments for cognitive dysfunction after stroke. The limited efficacy of interventions, as well as the growing interest in recovery from stroke sequelae, has led to development of integrated cognitive therapeutic strategies for cognitive rehabilitation, that is, the remediation or alleviation of cognitive deficits resulting from neurological damage after ischemic stroke [11].

Computer-assisted cognitive rehabilitation (CACR) and cholinesterase inhibitors (ChEIs), either individually or as part of an integrated cognitive therapy, are the main clinical treatments for cognitive dysfunction to date. Although some studies have reported the effects of CACR and ChEIs on cognitive function [12, 13], there remains a paucity of studies concerning the effect of both CACR and ChEIs on hippocampal FC changes in cognitively impaired ischemic stroke patients. Therefore, we examined the incidence of hippocampal FC abnormalities in cognitively impaired ischemic stroke patients compared to healthy controls (HC) using the Mini-Mental State Examination (MMSE) and Wechsler Memory Scale (WMS). Additionally, we investigated whether these abnormal hippocampal FCs could be modulated by integrated cognitive therapy.

## 2. Materials and Methods

### 2.1. Subjects

This study was approved by the Medical Ethics Committee of Fujian University of Traditional Chinese Medicine (Fujian, China). From September 2013 to March 2014, 36 right-handed subjects were enrolled after providing written informed consent, including 18 ischemic stroke patients with cognitive dysfunction and 18 HCs. All included ischemic stroke patients were being treated at the Fujian University of Traditional Chinese Medicine Rehabilitation Hospital. HC subjects were recruited from the local community and matched by age, sex, education, and handedness.

All participating ischemic stroke patients fulfilled the following inclusion criteria: (i) having had a clinically diagnosed (by computed tomography or MRI) first ischemic stroke involving unilateral infarction of the basal ganglia without significant hemorrhagic transformation within 6 months of study inclusion; (ii) aged 40–75 years; (iii) completed at least 6 years of education; (iv) had a MMSE score ≤24; (v) had clinically verified memory deficits following stroke (verified by WMS); and (vi) had conscious and stable physical condition. Exclusion criteria were: (i) severe hearing and visual problems inhibiting completion of rehabilitation training and assessment; (ii) pregnancy; (iii) very severe neglect; (iv) complete hemianopsia in one half of their visual field (with hemianopic reading disorder); and (v) history of cognitive decline prior to stroke. Inclusion criteria for HC subjects were as follows: (i) no neurological deficits, including visual or hearing loss; (iii) no positive sign in the neurological exam; (iv) no cognitive complaints; and (v) an MMSE score ≥28. In order to ensure data quality, only patients with an uninjured hippocampus and no other contraindications via MRI were enrolled in this study. Detailed demographic and clinical data of all participants are presented in Table 1.

### 2.2. Experimental Design

#### 2.2.1. Integrated Cognitive Therapy

All ischemic stroke patients received CACR training from RehaCom software, which consists of training programs related to attention, memory, and executive functions. Patients in the CACR program trained under the supervision of physiotherapists 30 min/day, 5 days/week, for a total of 60 times over 3 months; CACR was administered by the same personnel each day. In combination with CACR training, patients also received 5 mg/day donepezil (a ChEI) over 3 months. To rule out possible confounding factors, patients did not accept other concomitant medication except that which their doctor considered to be necessary for treatment of other health issues, such as hypertension and diabetes.

#### 2.2.2. Cognitive Function Test

Stroke subjects were administered the cognitive function test before (baseline) and after integrated cognitive therapy compared to HCs at baseline. The cognitive function test was also administered by the same personnel each day. The WMS and the MMSE were used as neuropsychological assessments. The WMS includes seven subtests which evaluate memory at different ages and provides a total “memory quotient” (MQ) that accounts for age-related mnemonic variability [14]. The MMSE assessed five categories, including orientation, registration, attention, calculation, recall, and language.

#### 2.2.3. fMRI Data

*(1) Data Acquisition*. MRI data were acquired using a 3.0-T Signa (GE) MR scanner. Each participant was asked to remain still, keep their eyes closed, and think of nothing in particular. A foam pillow was used to restrict head movement, and earplugs were used to reduce noise interference. fMRI was acquired axially using an echo-planar imaging (TR = 2000 ms, TE = 30 ms, flip angle = 90°, FOV = 240 × 240 mm, thickness = 4 mm, resolution = 64 × 64, gap = 1 mm, slices = 28). After the functional run, a high-resolution T1-weighted structural scan was obtained using a 3D-MP RAGE sequence (TR = 1900 ms, TE = 2.2 ms, TI = 900 ms, flip angle = 9°, resolution = 256 × 256, thickness = 1 mm, FOV = 240 × 240 mm, slices = 176).

*(2) Data Preprocessing*. Preprocessing of fMRI data was performed using a statistical parametric mapping software package (SPM8, http://www.fil.ion.ucl.ac.uk/spm/). The data were preprocessed by removing the first 10 time points for the signal equilibrium (total of 160 time points) and participants' adaptation to the environment. The remaining time points were used for FC analyses.

First, image data underwent slice-timing correction and realignment for head motions. No participants had head motion more than 1.5 mm of translation in any of the *x*, *y*, or *z* directions or greater than 1.5° of rotation. The individual structural image was coregistered to the mean functional image after motion correction using linear transformation. The transformed structural images were then segmented into gray matter (GM), white matter (WM), and cerebrospinal fluid (CSF) by using a unified segmentation algorithm [15]. Then, the motion corrected functional volumes were spatially normalized to the Montreal Neurological Institute space and resliced to 2 × 2 × 2 mm voxels using the normalization parameters estimated during unified segmentation. Subsequently, the resulting images were smoothed with a Gaussian kernel of 4 × 4 × 4 mm (full-width half-maximum, FWHM). Finally, the resulting fMRI data were band-pass filtered (0.01 <*f*< 0.08 Hz) to reduce low-frequency drift and high frequency physiological respiratory and cardiac noise.

To further reduce the effects of confounding factors, we also used a multiple regression process to further remove the effects of head motion and other possible sources of artifacts: (i) whole-brain signal averaged over the entire brain, (ii) WM signal averaged from WM regions of interest [ROIs], (iii) CSF signal averaged from CSF ROIs, and (iv) six motion parameters.

*(3) fMRI Analysis of Resting-State FC*. Using the Automated Anatomical Labeling software template, the bilateral hippocampus was chosen as the ROI [16]. All fMRI data were assessed using SPM8 software. Reference and voxel time-series data were evaluated by Pearson's correlation analysis. Correlation coefficients were transformed into *z*-scores using Fisher's *r*-to-*z* transformation to improve normality. A one-sample *t*-test was performed to identify brain regions that significantly correlated with the seed region in each group under a combined threshold of *P* < 0.05 and cluster size ≥40 voxels. This yielded a corrected threshold of *P* < 0.01 using the AlphaSim program in AFNI software (FWHM = 4 mm, with mask).

Individual *z*-values were also entered into a random effects two-sample *t*-test to identify brain regions with significantly different hippocampal FC between 18 stroke and 18 HC subjects; a paired Student's *t*-test was used to identify the regions with significant hippocampal FC differences before and after therapy. Voxels survived a corrected threshold of *P* < 0.05 and cluster size ≥40 voxels. This yielded a corrected threshold of *P* < 0.01 using AlphaSim (FWHM = 4 mm, with mask). Pearson's correlation analysis between *z*-values and neuropsychological scores was performed to explore whether hippocampal FC varies with disease progression and cognitive performance in stroke patients. First, averaged *z*-values of each cluster with significant group differences were extracted. Then, Pearson's correlative analyses were performed to examine relationships between *z*-values and neuropsychological performance.

#### 2.2.4. Statistical Analysis

All statistical analyses were performed using SPSS version 18.0 (Chicago, IL, USA) unless specified otherwise. All neuropsychological assessment data were expressed as mean ± standard deviation. Statistical differences in MMSE and WMS measurement and enumeration data were analyzed using a Student's *t*-test and Chi-square test, respectively. Intragroup comparisons were assessed by paired Student's *t*-test. Pearson's correlative analysis was performed using SPSS. A *P* < 0.05 was considered statistically significant.

## 3. Results

Overall, 15 stroke and 18 HC subjects were used for final data analysis, as three stroke patients with head motion more than 2 mm of translation were excluded from this study. Of the included stroke patients, nine had left-side and six had right-side unilateral basal ganglia infarctions. No significant differences between gender, age, and years of education were noted between the two groups; however, MMSE and WMS scores were significantly different (*P* < 0.05; Tables 1 and 2).

### 3.1. Hippocampal FC Patterns in Ischemic Stroke versus HC Subjects at Resting-State

**Figure 1:**
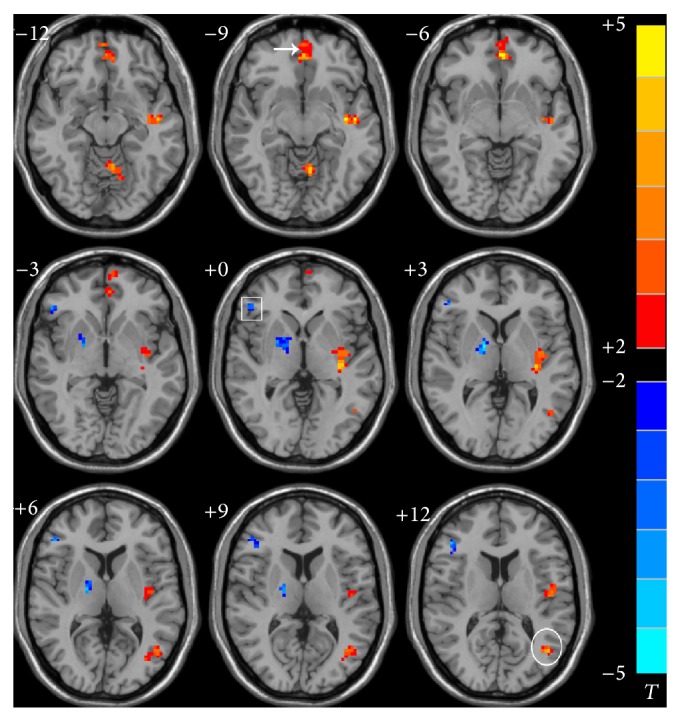
Abnormal FC pattern of the left hippocampus in the ischemic stroke patients compared to HC in resting state. The voxels with hot color represent HFC positive functional connectivity, and the voxels with cold color represent HFC negative functional connectivity. (*P* < 0.05 and cluster size >=40 voxels, multiple comparisons corrected). Left is the right; right is the left. Square: inferior frontal gyrus. Arrow: medial frontal gyrus. Circle: middle temporal gyrus.

**Figure 2:**
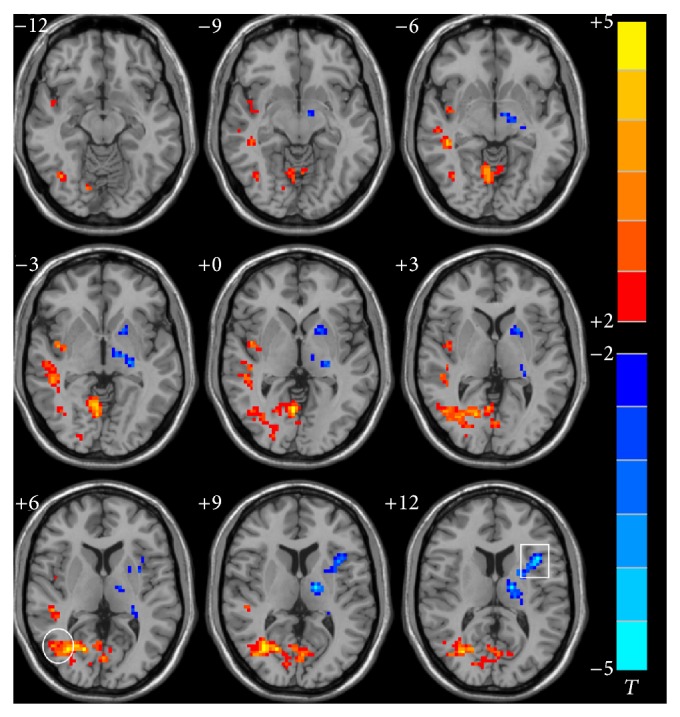
Abnormal FC pattern of the right hippocampus in the ischemic stroke patients compared to HC in resting state. The voxels with hot color represent HFC positive functional connectivity, and the voxels with cold color represent HFC negative functional connectivity. (*P* < 0.05 and cluster size >=40 voxels, multiple comparisons corrected). Left is the right; right is the left. Square: inferior frontal gyrus. Circle: middle temporal gyrus.

Left and right hippocampal FC maps of ischemic stroke versus HC subjects in the resting-state are shown in Figures 1 and 2, respectively. Compared to HCs, cognitively impaired stroke patients showed an increased resting-state FC of the right hippocampus with the right medial and superior temporal gyrus and insular cortex and increased left hippocampal resting-state FC with the left medial and temporal frontal gyrus, left anterior and posterior cerebellum, and insular cortex. A decrease in resting-state FC of the right hippocampus was found with the left superior frontal and temporal gyrus and left thalamus, as well as in the left hippocampus with the right inferior frontal gyrus and thalamus. Detailed Talairach coordinates of these regions are presented in Table 3.

### 3.2. Significant Differences in Hippocampal FC Connectivity after Integrated Cognitive Therapy

**Figure 3:**
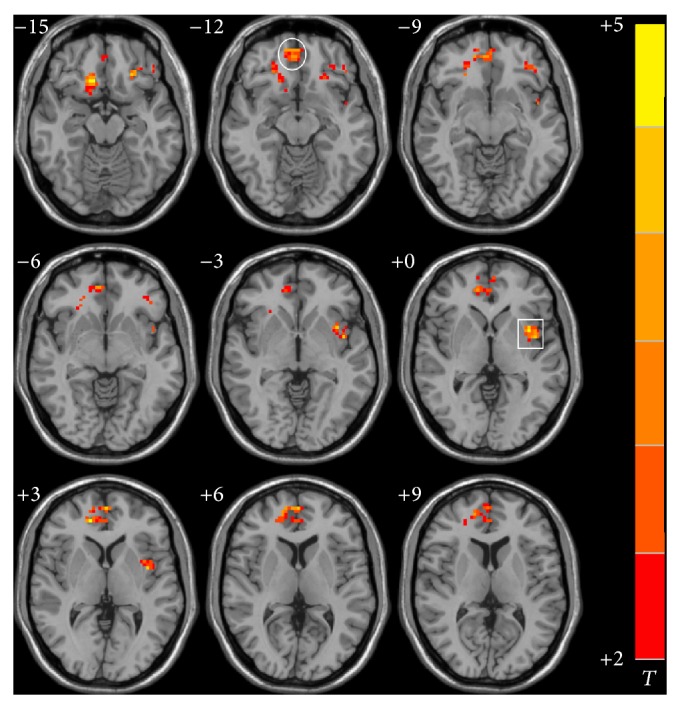
Significant differences in functional connectivity of left hippocampus in the ischemic stroke patients after the integrated cognitive therapy. (*P* < 0.05 and cluster size >=40 voxels, multiple comparisons corrected). Left is the right; right is the left. Square: insular cortex. Circle: medial frontal gyrus.

**Figure 4:**
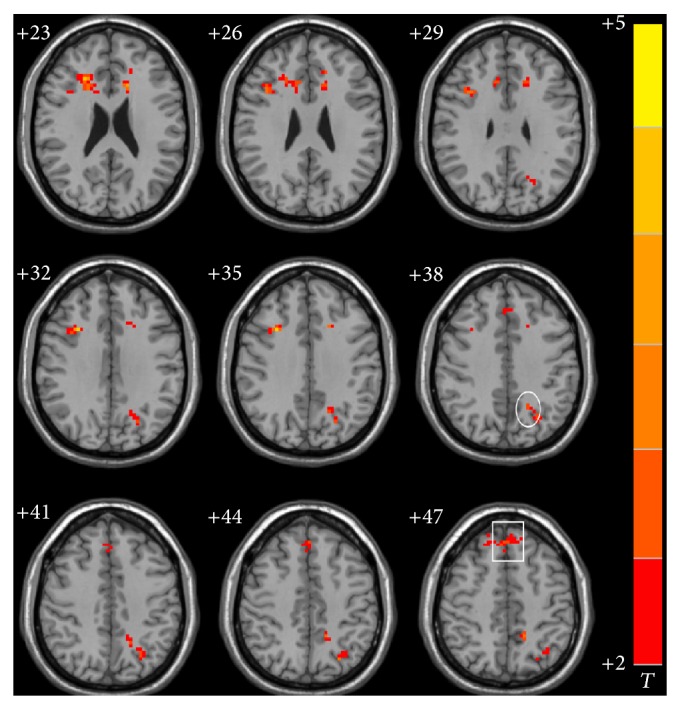
Significant differences in functional connectivity of right hippocampus in the ischemic stroke patients after the integrated cognitive therapy (*P* < 0.05 and cluster size >=40 voxels., multiple comparisons corrected). Left is the right; right is the left. Square: superior frontal gyrus. Circle: precuneus.

Left and right hippocampal FC maps for cognitively impaired ischemic stroke patients after integrated cognitive therapy are shown in Figures 3 and 4, respectively. Following therapy, stroke patients showed increased resting-state FC of the left hippocampus with the left precuneus, insular cortex, and medial frontal gyrus and right inferior and medial frontal gyrus; an increased right hippocampal resting-state FC was found with the right medial and superior frontal gyrus and left inferior frontal gyrus, posterior cerebellum, and precuneus. Detailed Talairach coordinates of these regions are presented in Table 4.

### 3.3. Correlations between Hippocampal FC and Neuropsychological Measures after Integrated Cognitive Therapy

A significant improvement in the MQ and left hippocampal resting-state FC with the right frontal lobe (*r* = 0.65; *P* < 0.05) and left precuneus (*r* = 0.55; *P* < 0.05), as well as right hippocampal resting-state FC with the right frontal lobe (*r* = 0.71; *P* < 0.05) and left precuneus (*r* = 0.61; *P* < 0.05), was found in cognitively impaired ischemic stroke patients.

## 4. Discussion

Cognitive dysfunction is an area of great importance in neuroscience research. Improving cognition and defining mechanism(s) of recovery in cognitive training are of utmost importance to increase the quality of life of stroke patients. In particular, investigating brain FC represents a potentially useful and noninvasive way in which to investigate the strength of widely distributed neural networks in HCs and patients with various neurological or psychiatric disorders [16]. CACR has been widely used since its introduction in 1986 [17] and has become a standard in cognitive rehabilitation, as it provides a battery of standardized tasks with immediate feedback. Thus, it is extremely useful when conducting patient follow-up exams and performing clinical studies [2].

Previous studies have reported significant cognitive improvements in stroke patients using CACR [18–20]. Further studies have indicated that concomitant use of CACR with other conventional treatments may confer additional improvements to cognitively impaired stroke patients [2]. For example, a clinical trial has demonstrated that ChEIs, such as donepezil, positively affect cognition and cause FC changes associated with cognitive improvement [12]. Therefore, we investigated whether concomitant use of ChEIs and CACR could modulate abnormal hippocampal FCs in cognitively impaired ischemic stroke patients after 3 months.

*Abnormal Hippocampal FC in Stroke Patients*. In the resting-state, we found abnormal hippocampal FCs associated with the temporal regions (thalamus, medial, and superior temporal gyrus), insular cortex, cerebellum, and prefrontal cortex (inferior, medial, and superior frontal gyrus) in cognitively impaired ischemic stroke patients compared to HCs. Our results are in agreement with a recent study showing significant alterations in the resting-state network of brains of stroke patients (RSN), including increased brain activity in the frontal, frontotemporal, and default-mode networks (DMN) [21]. In addition, the brain regions that showed increased activity in the resting-state are related to cognitive dysfunction in stroke patients, especially executive, attentional, and memory function.

Neuroanatomically, both prefrontal and medial temporal cortices are involved in memory processing [22]. The prefrontal cortex plays a crucial role in both mental holding and manipulation of information within working memory [23, 24], as well as regulation of cognitive function [25]. This brain region also performs short-term storage of input data and is involved in controlling working memory, which is closely related to cognitive processes such as language learning, long-term memory, and executive function [26, 27]. The medial temporal lobes are key to the formation of new memories and integration of various aspects of memories [22]. For example, a previous study demonstrated that the medial temporal lobe is involved in sequence memory [28]. Studies have also shown that a series of parallel temporal-diencephalic pathways exist between the hippocampus and anterior thalamic nucleus that function in a mutually beneficial manner, both directly and indirectly. These multiple hippocampal-anterior thalamic interconnections are both critical for human episodic memory and rodent event memory [29].

DMN regions often show negative activation when participants perform cognitive tasks, and the degree of inactivation predicts performance [30]. DMN areas include the medial posterior cortex, specifically the posterior cingulate and medial frontal cortices, the precuneus, and bilateral inferior parietal, and posterior temporal areas around the temporoparietal junction [31]. The precuneus is an important component of the DMN involved in the interwoven network of neural correlates of self-consciousness, mostly engaging in self-related mental representations during rest [32]. The insular cortex has been implicated in emotion processing and regulation, as well as in other cognitive processes. Moreover, a previous study has emphasized that the right insular and frontal cortices represent network hubs mediating between the central executive network and DMN, and, thus, may be critically involved in cognitively demanding tasks [33]. In addition, previous studies have also shown that, besides motor function, the cerebellum also plays a critical role in cognitive control [34, 35] and has been shown to be activated during cognitive tasks [36, 37]. Because these brain areas are closely associated with cognitive function, we presumed that abnormal FC within these brain areas might lead to cognitive dysfunction in patients with stroke.

Furthermore, previous studies have shown that RSN changes in stroke patients could be interpreted as brain dysfunction due to stroke. When these brain regions are injured after stroke, they compensate for damaged areas by shifting FC to favor the use of stronger, unaffected regions. Therefore, patients showing a larger shift in FC have better cognitive performance [21]. This result is consistent with a previous report that shows that increased connectivity recruits more neural resources from unaffected brain regions to compensate for cognitive losses [38]. Therefore, we concluded that an increased hippocampal FC might be considered a recruitment of additional neural resources to compensate for declines in cognitive function. Moreover, increased hippocampal FC with the prefontal cortex and DMN might represent major compensatory mechanisms.

*Effect of Integrated Cognitive Therapy on Hippocampal FC*. Our main hypothesis was that integrated cognitive therapy would improve the hippocampal FC network and significantly correlate with cognitive improvement. As we found in this study, after treatment, stroke patients showed significantly increased hippocampal FC mainly within the prefrontal gyrus and DMN (Table 4; Figures 3 and 4). The fact that prefontal and DMN connectivity were stronger in stroke patients after cognitive recovery also supports the hypothesis that increased hippocampal FC within these regions was the main compensatory mechanism in cognitively impaired stroke patients. To further examine whether these alterations correlated with cognitive performance, Pearson's correlative analyses were performed; correlations between hippocampal FC and neuropsychological measures were statistically significant. This suggests that improvement of FC in stroke patients was closely related to cognitive recovery. Thus, we believe that FC enhancements may highly contribute to improved cognitive function to some extent, making FC a viable way in which to investigate the strength of distributed brain networks in both healthy subjects and patients with neurological and psychiatric disorders.

Interestingly, we previously found an increase in hippocampal FC within the frontal and left parietal lobes after CACR. In the present study, we also found increased FC in the precuneus and cerebellum after integrated cognitive therapy. This may be related to synergistic effects of combining CACR and donepezil therapies. However, further clinical studies are required to fully delineate the relationship between concomitant CACR and ChEI therapies with cognition. Therefore, we concluded that abnormal hippocampal FC in cognitively impaired ischemic stroke patients could be modulated by integrated cognitive therapy, and FC enhancements might be compensatory mechanisms for functional recovery after stroke.

## Limitations

Although cognitive dysfunction after ischemic stroke is influenced by many variables, we limited these variables by including patients who experienced a first unilateral ischemic stroke without significant hemorrhagic transformation. Unfortunately, we did not measure infarct depth and vascular territory because it requires further large-scale and more stringent inclusion criteria. In addition, stroke is a very complex disease and patients generally have different levels and types of underlying disease; some patients received other concomitant therapies to treat other healthy issues, including hypertension, diabetes, and hyperlipidemia. Although we limited these variables by excluding patients taking any drug associated with cognition, in addition to donepezil, it would be best to separately analyze those patients to verify whether their medication affects neuroimaging results and/or CACR performance.

The preliminary nature of this study and its small sample size meant that it was not possible to analyze changes in resting-state FC in subgroups of patients (e.g., stratified by sex, age, education level, or medication). In future studies, we intend to examine a larger sample size with more subgroups, including a ChEI group, CACR group, and stratification by medication, to provide a more detailed analysis of the effect of integrated cognitive therapy on hippocampal FC. Despite these limitations, we found that abnormal hippocampal FC exists in ischemic stroke patients. Furthermore, we demonstrated that altered hippocampal FC is associated with cognitive improvement and that increased FC between the hippocampus and prefrontal cortex might be a compensatory mechanism for functional recovery.

## Figures and Tables

**Table 1:** Clinical characteristics of the stroke group and the HC group.

Parameter	Stroke group (*n* = 18)	HC group (*n* = 18)	*P* value
Sex (male/female)	10/8	10/8	>0.99^#^
Age (years)	69.64 ± 6.88	68.07 ± 7.46	0.583^*^
Education (years)	10.26 ± 1.77	10.30 ± 2.05	0.958^*^
MMSE	13.26 ± 5.36	28.57 ± 0.65	<0.05^*^
WMS (Memory quotient)	77.43 ± 6.83		

Data are presented as mean ± SD.

MMSE: Mini-Mental State Examination; WMS: Wechsler Memory Scale.

^#^The *P* value was obtained using a Pearson *x* ^2^ two-tailed test.

^*^The *P* value was obtained by a two-sample two-tailed *t*-test.

**Table 2:** MMSE and Wechsler Memory Scale subtests at baseline and after treatment of the Ischemic stroke patients with cognitive dysfunction.

	Baseline	Posttreatment	*P* value
WMS subtsts			
Information	4.74 ± 0.37	4.96 ± 0.33	0.29^*^
Orientation	4.23 ± 0.14	4.29 ± 0.77	0.33^*^
Mental control	5.65 ± 0.38	7.33 ± 0.50	0.003^*^
Logical memory	6.71 ± 2.54	9.83 ± 2.97	0.001^*^
Digits forward and backward	8.21 ± 1.19	9.29 ± 2.41	0.020^*^
Visual reproduction	7.22 ± 1.3	10.23 ± 1.70	0.008^*^
Associate learning	6.9 ± 2.35	11.0 ± 1.39	0.000^*^
Memory quotient	77.65 ± 6.43	87 ± 7.98	0.006^*^
MMSE	13.26 ± 5.36	18.31 ± 4.39	<0.05^*^

Data are shown as mean ± SD.

^*^The *P* value was obtained by a two-sample two-tailed *t*-test.

**Table 3:** Regions showing abnormal HFC in the Ischemic stroke patients with cognitive dysfunction comparing to HC in resting state.

Anatomical regions	Side	Cluster size	Peak coordinates (MNI)			Peak *t*-score
			*X*	*Y*	*Z*	
Middle temporal gyrus^a^	R	82	33	−69	9	5.7958
Middle temporal gyrus^b^	L	60	−51	−9	18	5.2177
Superior temporal gyrus^a^	R	96	45	−33	−6	5.1406
Medial frontal gyrus^b^	L	53	0	48	−6	5.0744
Insular cortex^a^	L	48	−40	0	15	5.0512
Insular cortex^b^	R	42	42	−6	−3	4.4423
Cerebellum anterior lobe^b^	L	47	−6	−60	−9	4.7176
Cerebellum posterior lobe^b^	L	46	−39	−57	−30	3.7787
Thalamus^a^	L	35	−15	−12	9	−5.6475
Superior temporal gyrus^a^	L	30	−24	3	−27	−4.7568
Thalamus^b^	R	30	15	−3	3	−4.4357
Inferior frontal gyrus^b^	R	85	36	18	24	−4.0718
Superior frontal gyrus^a^	L	56	−33	27	30	−3.9030

^a^Changes about functional connectivity of the left hippocampus with all other brain voxels in this study. ^b^Changes about functional connectivity of the right hippocampus with all other brain voxels in this study.

*P* < 0.05, corrected for multiple comparison. R: right side; L: left side.

**Table 4:** Significant differences in functional connectivity of hippocampus in the ischemic stroke patients with cognitive dysfunction before and after the integrated cognitive therapy.

Anatomical regions	Side	Cluster size	Peak coordinates (MNI)			Peak *t*-score
			*X*	*Y*	*Z*	
Middle frontal gyrus^a^	R	36	24	27	24	5.1236
Cerebellum posterior lobe^a^	R	70	24	−78	−48	4.9730
Superior frontal gyrus^a^	R	66	6	36	60	4.7074
Inferior frontal gyrus^a^	L	40	−39	42	−12	4.6973
Precuneus^a^	L	47	−15	−48	51	4.0605
Precuneus^b^	L	67	−12	−69	39	3.6092
Medial frontal gyrus^b^	R	89	15	45	3	3.5207
Inferior frontal gyrus^b^	R	92	15	24	−15	3.3922
Middle frontal gyrus^b^	L	89	−24	30	−18	3.3803
Insular cortex^b^	L	38	−30	20	15	3.3789
Superior temporal gyrus^b^	L	35	−36	6	0	3.3758

^a^Changes about functional connectivity of the right hippocampus with all other brain voxels in this study. ^b^Changes about functional connectivity of the left hippocampus with all other brain voxels in this study.

*P* < 0.05, corrected for multiple comparison. R: right side; L: left side.
